# First description of a fatal equine infection with *Halicephalobus gingivalis* in Portugal. Relevance for public health

**DOI:** 10.1002/vms3.142

**Published:** 2019-01-22

**Authors:** Rute Noiva, Pedro Ruivo, Luís Madeira de Carvalho, Constança Fonseca, Miguel Fevereiro, Paulo Carvalho, Leonor Orge, Madalena Monteiro, Maria Conceição Peleteiro

**Affiliations:** ^1^ CIISA Interdisciplinary Centre of Research in Animal Health Faculdade de Medicina Veterinária Universidade de Lisboa Av. da Universidade Técnica Lisbon Portugal; ^2^ Integrated Masters Course of Veterinary Medicine Faculdade de Medicina Veterinária Universidade de Lisboa Av. da Universidade Técnica Lisbon Portugal; ^3^ HTS (Horses Therapy Services) Algueirão Portugal; ^4^ Instituto Nacional de Investigação Agrária e Veterinária (INIAV) Unidade Estratégica de Investigação e Serviços de Produção e Saúde Animal Av. da República Quinta do Marquês Oeiras Portugal

**Keywords:** *Halicephalobus gingivalis*, horse, parasitic meningoencephalitis, nephritis, granulomatous lymphadenitis, Portugal

## Abstract

*Halicephalobus gingivalis* is a small saprophytic rhabditid nematode, represented only by females with a typical rhabditoid oesophagus and one egg in the uterus, capable of infecting vertebrates. This opportunistic parasite present in the soil, manure and decaying humus, is thought to penetrate through previous injuries to the mouth, eyes and skin of horses and migrate to various organs. The brain is one such organ, where the females lay their eggs, leading to malacia and causing a sudden onset of neurological signs, such as anorexia, ataxia, urinary incontinence, blindness, decreased menace and tonal reflexes, tremors and aggressiveness. The disease is invariably fatal whenever brain lesions are present, and the diagnosis usually achieved only post‐mortem. The present work aims to describe the first case of infection by *H. gingivalis* ever reported in Portugal. An 8‐year old warmblood horse presented with an 8‐day history of progressive blindness involving the left eye, initially with normal pupillary reflexes, advancing to bilateral blindness and increasing deterioration in clinical condition. After euthanasia, the animal was submitted for necropsy. Organ samples were collected and fixed in 10% neutral buffered formalin for routine histopathology. A large mass was found in the left kidney corresponding to fibrous tissue heavily infiltrated with inflammatory cells and numerous nematodes. In the brain, multiple, bilateral and asymmetrical foci of malacia containing several rhabditoid nematodes, larvae and zygotes, and high numbers of inflammatory cells were found. The nematodes were identified as *H. gingivalis*. The clinical history, necropsy and histological findings presented constitute a typical case of *H. gingivalis* infection in a horse, never previously described in Portugal to the authors’ best knowledge. Humans can be infected by contact with contaminated manure, which makes this nematode a public health concern, especially for people living and/or working in close proximity to horses.

## Introduction

Neurological diseases in horses frequently go undiagnosed in Portugal. This is mostly a reflection of a deeply ingrained cultural unwillingness of the owners to accept necropsy procedures, even when recommended by the attending veterinarian. However, a number of diseases affecting these sports/leisure/companion animals can also affect humans, making diagnosis of such conditions particularly relevant.

The present work pertains to the first case of infection by *Halicephalobus gingivalis* ever reported in Portugal.

*H. gingivalis*, previously referred to as *Micronema deletrix or H. deletrix*, is a small 250–450 *μ*m by 15–25 *μ*m, free‐living saprophytic rhabditid nematode, represented only by females with a typical rhabditoid oesophagus and one egg in the uterus, capable of infecting vertebrates (Chitwood & Lichtenfels 1972; Johnson *et al*. 2001; Vasconcelos *et al*. 2007; Papadi *et al*. 2013; Eberhard 2014). This opportunistic parasite is commonly found in soil, manure, foul water or decaying humus (Johnson *et al*. 2001; Nadler *et al*. 2003). There are so far, <100 cases of halicephalobosis described in literature, mostly in horses (Rames *et al*. 1995; Anderson *et al*. 1998; Bröjer *et al*. 2000; Johnson *et al*. 2001; Nadler *et al*. 2003; Bryant *et al*. 2006; Vasconcelos *et al*. 2007) ‐ including ponies (Akagami *et al*. 2007) ‐, but also in zebras (Isaza *et al*. 2000), cattle (Enemark *et al*. 2016) and humans (Papadi *et al*. 2013; Anwar *et al*. 2015; Lim *et al*. 2015; Monoranu *et al*. 2015). *H. gingivalis* infection in horses has been reported in five continents, usually involving a wide range of ages (from colts to aging adults) and breeds, leading almost invariably, to a fatal outcome (Anderson *et al*. 1998; Lim *et al*. 2015; Monoranu *et al*. 2015). In Europe, the disease is reported in several countries, such as the United Kingdom, Belgium, Italy, Denmark, Spain, Iceland and Romania (Rames *et al*. 1995; Anderson *et al*. 1998; Bröjer *et al*. 2000; Johnson *et al*. 2001; Bryant *et al*. 2006; Akagami *et al*. 2007; Eydal *et al*. 2012; Gracia‐Calvo *et al*. 2014; Jung *et al*. 2014; Lim *et al*. 2015; Taulescu *et al*. 2015; Pintore *et al*. 2017). Owners seek veterinary assistance mostly due to unexpected and unexplained onset of neurological symptoms, such as anorexia, ataxia, urinary incontinence, blindness, decreased menace and tonal reflexes, tremors and aggressiveness (Rames *et al*. 1995; Anderson *et al*. 1998; Bröjer *et al*. 2000; Johnson *et al*. 2001; Bryant *et al*. 2006; Akagami *et al*. 2007; Vasconcelos *et al*. 2007). In a number of cases, the horses are reported to have suffered a prior injury to the mouth, eyes and skin (Rames *et al*. 1995; Anderson *et al*. 1998; Bröjer *et al*. 2000; Isaza *et al*. 2000; Eydal *et al*. 2012). Diagnosis is generally achieved only after death and necropsy. In the rare cases in which the disease has been correctly diagnosed *in vivo*, progression of the disease was quick (hours to a few weeks) and fatal, in spite of treatment (Rames *et al*. 1995; Isaza *et al*. 2000; Kinde *et al*. 2000). Human cases have been reported in various countries, including in North America, Canada (Hoogstraten & Young 1975) and USA (Shadduck *et al*. 1979; Ondrejka *et al*. 2010; Papadi *et al*. 2013; Anwar *et al*. 2015), Australia (Lim *et al*. 2015) and Europe, namely Germany (Monoranu *et al*. 2015), likewise affecting a wide range of patient ages (from 5 to 74 years) and usually involving clinical presentations of fever (which is rarely seen in affected horses (Bröjer *et al*. 2000; Bryant *et al*. 2006)), blindness, mental changes and lethargy. The reported outcome has always involved death of the patient.

## Materials and methods

In August 2016, an 8‐year old warmblood horse, never having moved out of the country where it was born, was submitted for clinical evaluation due to an 8‐day history of progressive blindness involving the left eye, initially with normal pupillary reflexes, which soon progressed to bilateral blindness. Acute onset of other neurological symptoms followed, such as ataxia difficulty in food prehension and mastication and dysphagia. Blood cell counts, liver and urinary enzyme tests were within normal limits. Serology was negative for West Nile Virus (WNV) (IgM and IgG) and for equine herpesvirus type 1 (EHV‐1) and type 4 (EHV‐4), by ELISA. Treatment with 12 mg of dexamethasone (intravenous, SID), 0.5 g kg^‐1^ DMSO as a 10% solution (intravenous, SID), oxytetracycline 6.6 mg kg^‐1^ (intravenous, SID) and intramuscular vitamin B resulted in some temporary relief although blindness and ataxia persisted. Within only a few days after the treatment started, the animal's clinical condition deteriorated, and euthanasia was requested by the owners. The animal was then submitted for necropsy and routine histopathological examination. Organ samples were collected and fixed in 10% neutral buffered formalin for routine histopathology. Microscopic observation was based on 3 *μ*m Haematoxylin and Eosin (H&E) stained sections. Cerebrospinal fluid was collected for virology.

## Results

**Figure 1:**
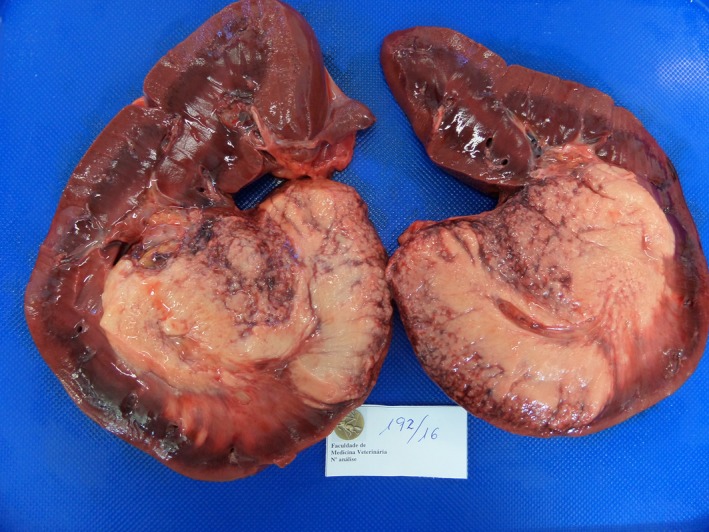
Left kidney cut section largely occupied by a yellowish firm tissue, with small confluent granules at the periphery, sometimes with acinar pattern.

**Figure 2:**
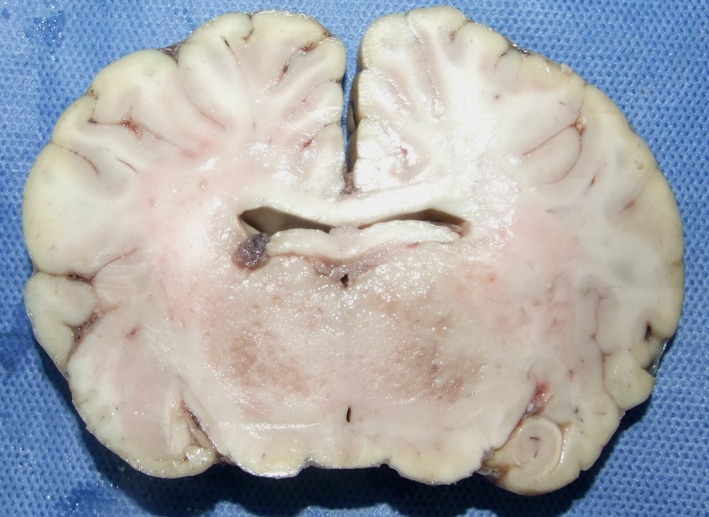
Brain section at the level of the thalamus, showing small foci of malacia.

At necropsy, the body presented good overall condition. The significant lesions were: mild enlargement of left kidney due to a large mass of yellowish firm tissue, conspicuous only when the organ was cut, which occupied approximately more than 50% of its volume, with small confluent granules, sometimes with acinar pattern at the periphery (Fig. 1); enlargement and congestion of the homolateral renal lymph node and, in the brain, multiple, bilateral and asymmetrical foci of malacia that were particularly evident in the thalamus (Fig. 2). Other lesions pertained to the small intestine, which showed mild congestion with abundant and fluid content.

Attempts to isolate WNV in cultures of Vero E6 and EHV in RK13 and equine fetal kidney cells were negative after two passages. Detection of EHV by conventional PCR (VanDevanter *et al*. 1996) and qPCR (Fortier *et al*. 2009), as well as detection of WNV by RT‐qPCR (Barros *et al*. 2013), were negative in peripheral blood mononuclear cells, brain tissue and cerebrospinal fluid.

**Figure 3:**
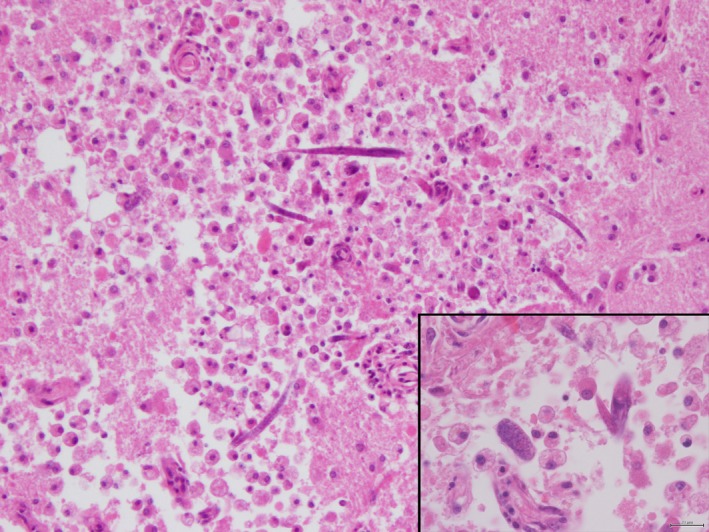
Brain microscopy. Thalamus. Numerous gitter cells surround multiple parasitic forms in an area of extensive necrosis. Insert – Embryonated zygote and part of a larva. (Hematoxylin&Eosin)

**Figure 4:**
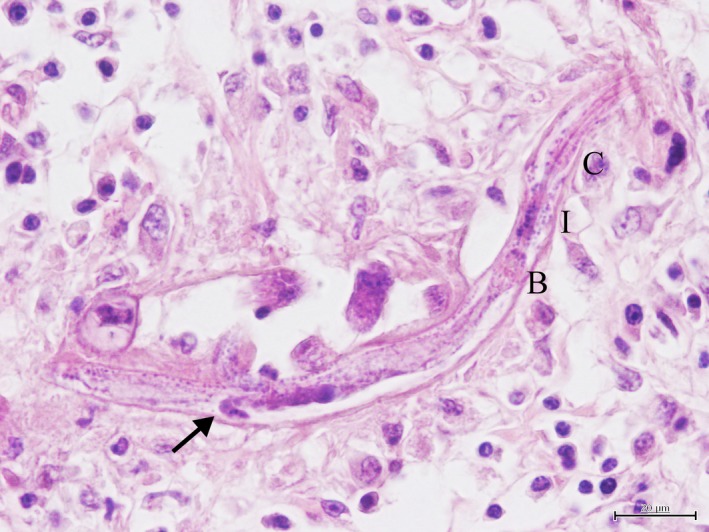
Brain microscopy. Thalamus. Adult female showing the rhabditoid oesophagus with a corpus (C), isthmus (I) and bulb (B). No egg can be seen in the uterus, although the reflected ovary is quite clear (arrow). (Hematoxylin&Eosin)

The brain histology showed ill‐defined necrotic areas in the thalamus containing several rhabditoid nematodes, larvae and zygotes, high numbers of gitter cells, multinucleated, sometimes Langhans‐type, giant cells, spheroids and perivascular inflammatory cells (mainly lymphocytes and occasionally eosinophils) (Fig. 3). Eggs were seen loose in the necrotic brain tissue, measuring on average 30 × 12 *μ*m (Fig. 3, insert). The same lesions, although less severe, were observed in the midbrain, cerebellum and occipital cerebral cortex. A mild multifocal accumulation of mononucleated inflammatory cells was present in the meninges. Complete female adults could be seen, measuring on average, 200 *μ*m by 15 *μ*m (measurements were taken with a DP23 Olympus Digital Camera software), with a mean width of 7 *μ*m, pointed tail, and clearly featuring typical internal structures such as the rhabditoid oesophagus with a corpus, an isthmus (10 *μ*m mean width) and a bulb and the dorsally reflected ovary (Fig. 4).

**Figure 5:**
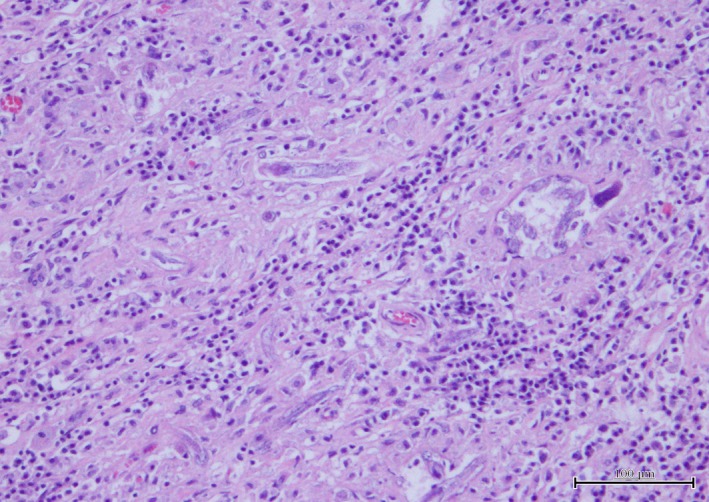
Left kidney microscopy. Various parasitic forms can be seen in the fibrous tissue heavily infiltrated with inflammatory cells. (Hematoxylin&Eosin)

Histopathology of the kidney revealed that the yellow tissue corresponded to fibrous connective tissue which largely replaced the parenchyma, heavily infiltrated with inflammatory cells, mainly mononucleated (plasma cells, lymphocytes and macrophages), with frequent multinucleated cells. Within this tissue numerous sections of parasites were identified (Fig. 5), occasionally featuring the typical rhabditoid oesophagus. Multiple embryonated zygotes could also be seen, especially where the parasites were more numerous.

The renal lymph node showed lymphoid hyperplasia and various haemorrhagic foci. Small numbers of parasites could also be seen, including embryonated zygotes, either deep in the cortex or closer to the surface.

The final diagnosis was of severe chronic parasitic granulomatous necrotizing meningoencephalitis and nephritis, with granulomatous lymphadenitis of the renal lymph node, caused by infection with rhabditiform nematodes of the genus *Halicephalobus*.

## Discussion

The clinical history, necropsy and histological findings hereby presented constitute a typical case of *H. gingivalis* infection in a horse, never previously described in Portugal to the authors’ best knowledge.

The gross and microscopic lesions described in this case are quite similar to what was reported by other authors in the various countries where the disease has been identified (Bryant *et al*. 2006; Eydal *et al*. 2012; Isaza *et al*. 2000; Johnson *et al*. 2001; Pearce *et al*. 2001; Rames *et al*. 1995; Taulescu *et al*. 2015). The identification of the parasite as *H. gingivalis* is particularly facilitated due to the presence of the characteristic rhabditoid oesophagus and the dorsally reflected ovary in the adult females (Chitwood & Lichtenfels 1972; Gardiner & Poynton 1999). Although other parasites can be found associated with lesions of the nervous system (*Hypoderma lineatum, H. bovis, Draschia megastoma, Strongylus vulgaris* and the hydatic cysts from *Echinococcus granulosus)* the lesions produced and the parasite's morphology are highly specific, allowing for an undoubtable diagnosis of halicephalobosis (van der Kolk & Kroeze 2013).

In the present case, no molecular characterization of the parasite was performed in order to confirm unequivocally the parasite's classification, as other authors have done (Nadler *et al*. 2003; Rodriguez *et al*. 2013; Pintore *et al*. 2017). However, the geographic distribution of the parasite in Europe and the lesions described make it quite unlikely that any other species may be involved.

Regarding preferred locations for the parasite, the brain and the kidney are generally described as the most significantly affected organs, followed by the oral and nasal cavities, lymph nodes, spinal cord, adrenal glands, heart, stomach, liver, peripheral nerve ganglia, bones and joints (Rames *et al*. 1995; Isaza *et al*. 2000; Johnson *et al*. 2001; Pearce *et al*. 2001; Bryant *et al*. 2006; Eydal *et al*. 2012). Larvae and eggs or zygotes are most commonly found in the infected tissues, with rare adults present (Anderson *et al*. 1998; Bröjer *et al*. 2000; Isaza *et al*. 2000; Johnson *et al*. 2001; Bryant *et al*. 2006; Akagami *et al*. 2007; Vasconcelos *et al*. 2007; Muller *et al*. 2008). Adult forms are invariably females described with a typical rhabditoid oesophagus, with a body, isthmus and a dilated bulb and only one egg in the uterus. They are assumed to reproduce by parthenogenesis within the host, which most likely contributes to the massive numbers of nematodes often found in tissues and swift clinical evolution towards death (Rames *et al*. 1995; Isaza *et al*. 2000; Kinde *et al*. 2000; Johnson *et al*. 2001; Nadler *et al*. 2003; Bryant *et al*. 2006; Eberhard 2014).

It still remains unclear how animals become infected with this facultative parasite but the most widely accepted hypothesis states that it occurs after contamination of exposed wounds (often oral or nasal, based on the common infection of these sites) and later spread through tissues and into the bloodstream and lymph, from which they could reach the two most commonly affected sites: the brain and the kidneys (Anderson *et al*. 1998; Bröjer *et al*. 2000; Bryant *et al*. 2006; Akagami *et al*. 2007). However, an obvious entry lesion may not be apparent at the time of diagnosis as was the case of the horse described here. Invasion of the brain through the optic stalk (after contamination of ocular wounds) has also been pointed as a possible pathway of infection, in those cases in which the eye is the primary site of lesion/infection (Rames *et al*. 1995). Ingestion of contaminated colostrum is yet another possible entryway, as suspected in an anecdotal report involving a mare with mastitis and her foal, which later developed encephalitis (Wilkins *et al*. 2001).

Treatment of *H. gingivalis* infections in animals is usually unsuccessful, among other things due to the inability of anthelmintic drugs to cross the blood–brain barrier and penetrate the granulomatous lesions in the brain (Papadi *et al*. 2013). There have been only two reports of successful treatment of localized, extra‐central nervous system *Halicephalobus* sp. infections in horses, with ivermectin alone or with diethylcarbamazine (Dunn *et al*. 1993; Schmitz & Chaffin 2004).

However, considering the severity of the lesions, it can be anticipated that the damage caused by the infection could be irreversible, in spite of the efficacy of anthelmintic drugs. As with horses, the cases described in humans were invariably fatal (Papadi *et al*. 2013; Anwar *et al*. 2015; Lim *et al*. 2015; Monoranu *et al*. 2015). Gross lesions included diffuse changes such as oedema or hyperaemia of brain parenchyma and dullness of meninges. Microscopically, all cases report brain necrosis and meningoencephalitis with a mixed eosinophil rich inflammatory infiltrate.

Although *H. gingivalis* infection is not contagious, the fact that humans can be infected by contaminated manure makes this nematode a public health concern, especially when people living and/or working in close proximity to horses are considered. As parasitic neurohelminthosis are uncommon, physicians do not routinely consider parasitic infections in the differential diagnosis of meningoencephalitis (Papadi *et al*. 2013). With no easy screening or confirmatory tests for parasitic meningoencephalitis, a definitive diagnosis of halicephalobosis is difficult to reach in the absence of accessible lesions for biopsy (Papadi *et al*. 2013). Although extremely uncommon, rapidly progressing neurologic cases, especially those associated with previous, chronically infected wounds in horse handlers/keepers and other people in reportedly contaminated environments, should include a differential diagnosis of halicephalobosis, especially if other possible infections have been ruled out.

Considering that the “preferentially” targeted host is the horse, this makes the veterinarian's function of primary importance in detecting possible foci of contamination and monitoring parasite presence in the environment, particularly through the correct diagnosis of possible cases in horses exhibiting unexplained neurological signs. As Public Health professionals, veterinarians should also alert physicians and people closer to or engaged in horse production and its environments, to the risk of this disease, particularly farmers, horse handlers/keepers and especially children, who frequently play in potentially contaminated soil and mud.

## Source of Funding

This work was supported by Grants CIISA, from CIISA Project UID/CVT/00276/2013, from Fundação para a Ciência e Tecnologia (FCT), Portugal.

## Conflict of Interest

None.

## Ethical Statement

The authors confirm that the ethical policies of the journal, as noted on the journal's author guidelines page, have been adhered to. No ethical approval was required as this is a case report with no original research data. Informed consent was obtained from the client for the publication of this case report.

## Contribution

Rute Noiva was chiefly involved in the drafting of the article, with various critical revisions and additions made by Luís Madeira de Carvalho, Constança Fonseca, Miguel Fevereiro, Paulo Carvalho, Leonor Orge, Madalena Monteiro and Maria Conceição Peleteiro. Pedro Ruivo, Luís Madeira de Carvalho, Constança Fonseca, Miguel Fevereiro, Paulo Carvalho, Leonor Orge, Madalena Monteiro and Maria Conceição Peleteiro were involved in the clinical and/or laboratorial examination of the animal as well as in the investigation and emission of the final diagnosis. All authors revised and approved the final version to be published.
